# A Diagnostic Dilemma: Clindamycin-Resistant Group A Streptococcal Toxic Shock Syndrome Leading to Acute Respiratory Distress Syndrome (ARDS) With Multiple Possible Sources

**DOI:** 10.7759/cureus.58212

**Published:** 2024-04-13

**Authors:** Donald Dennis, Sagar Kumar, Huma Akta, Brian Fouty, Kane Schaphorst

**Affiliations:** 1 Department of Internal Medicine, University of South Alabama, Mobile, USA; 2 Department of Pulmonary and Critical Care, University of South Alabama, Mobile, USA; 3 Department of Internal Medicine, Dow University of Health Sciences, Civil Hospital Karachi, Karachi, PAK

**Keywords:** acute respiratory distress syndrome (ards), septic shock (ss), gynecologic infections, group a streptococcal disease, streptococcal toxic shock syndrome, prone ventilation, streptococcal, linezolid, clindamycin, stss

## Abstract

Group A Streptococcal (GAS) infections can potentially progress into streptococcal toxic shock syndrome (STSS) with multiorgan failure. Even with a benign presentation, GAS can rapidly lead to fatal necrotizing infections. While myositis and cutaneous infections are the typical initial presentation of STSS, genitourinary infections are a less common source. This report presents a case of a previously healthy woman with the chief complaint of ankle pain who subsequently developed streptococcal toxic shock syndrome and multiorgan failure from a Group A streptococcus infection of the genitourinary tract. She was treated with antibiotics and medical management for her septic shock and required prone ventilation for her acute respiratory distress syndrome (ARDS) but eventually recovered without surgery. This case highlights the importance of recognizing unusual presentations of Group A Strep infections, which have the potential to lead to rapid deterioration in patients. Also described are antibiotic and ventilator strategies that can be used to treat these severe systemic infections.

## Introduction

Group A Streptococcus (GAS) is a Gram-positive bacterium that can cause a spectrum of diseases, including but not limited to pharyngitis, cellulitis, rheumatic fever, post-streptococcal glomerulonephritis, and streptococcal toxic shock syndrome (STSS). STSS is characterized by positive cultures for GAS in typically sterile locations, such as blood, which is accompanied by hypotension and multiorgan failure. This is believed to be a result of a profound inflammatory response secondary to superantigens and endotoxins produced by GAS [1]. Acute respiratory distress syndrome (ARDS) has been reported to be present in >50% of cases of STSS and should be considered in the setting of bilateral airspace disease as lung protective ventilatory strategies have proven mortality benefits in these patients [2,3]. Myositis or necrotizing fasciitis is commonly implicated in the etiology of this rare syndrome, whereas genitourinary infections in non-pregnant females have rarely been reported source [2,4].

We present a case of STSS in an otherwise healthy, immunocompetent, and young female who presented with septic shock, multiorgan failure, and ARDS in the setting of a pelvic infection and ankle swelling. The patient was successfully treated with antibiotics and supportive care that included lung-protective ventilation. This is an interesting and unique case in that while pelvic infections are not the typical source of Group A STSS; the literature is lacking on documented cases of pelvic infections leading to Group A STSS in the presence of other concurrent and confounding inflammatory processes. In this case, a fractured ankle with surrounding edema complicated the diagnostic approach.

## Case presentation

A 35-year-old female with a history of sexually transmitted infections (STI) presented to the Emergency Department (ED) with nine days of left ankle pain and swelling along with one day of shortness of breath and nonproductive cough. She reported associated chills, night sweats, and nausea. The patient had been seen in the ED five days prior with a swollen painful ankle in the absence of any known recent injury or skin breaks and without any systemic signs or symptoms. She was discharged at that time with a diagnosis of an ankle sprain.

Upon this second presentation, the patient was tachycardic to 130 beats per minute, afebrile, normotensive, and maintained adequate oxygen saturation on room air. On physical examination, she was in moderate distress due to ankle pain and the left ankle was erythematous and swollen with limited mobility due to pain. The pelvic exam revealed significant right labial swelling and tenderness with a cervical discharge. Pertinent labs on admission are presented in Table 1 and indicate leukocytosis, renal insufficiency, and possible hepatic injury.

**Table 1:** Admission laboratory values mg/dL: milligrams per deciliter, mMol/L: millimoles per liter, mm/hr: millimeters per hour, x10^3^/mcL: 10^3^ per microliter (referring to cell counts), g/dL: grams per deciliter, BUN: blood urea nitrogen; ALT: alanine aminotransferase, AST: alanine aspartate aminotransferase, ALP: alkaline phosphatase, CRP: C-reactive protein, ESR: erythrocyte sedimentation rate, WBC: white blood cell count, INR: international normalized ratio

Lab Name	Lab Value	Reference Range
BUN	20 mg/dL	7 - 18 mg/dl
Creatinine	2.05 mg/dL	0.53 - 1.02 mg/dl
Sodium	134 mMol/L	136 - 145 mMol/L
ALT	87 units	19 - 63
AST	79 units	15 - 37 units
ALP	317 units	45 - 117 units
Total Bilirubin	2.9mg/dl	0.0 - 1.0 mg/dl
CRP	37.9 mg/dl	<0.03 mg/dl
ESR	92 mm/hr	20 mm/hr
Lactate	1.8 mMol/L	<2 mMol/L
WBC	33 x10^3^/mcL	4.0 x10^3^ - 11.0x10^3^/mcL
Hemoglobin	9.7 g/dL	12.0 - 15.0 g/dl
Platelet count	185 x10^3^/mcL	150 - 450 x 10^3^/mcL
INR	1.30	0.8 - 1.2

**Figure 1:**
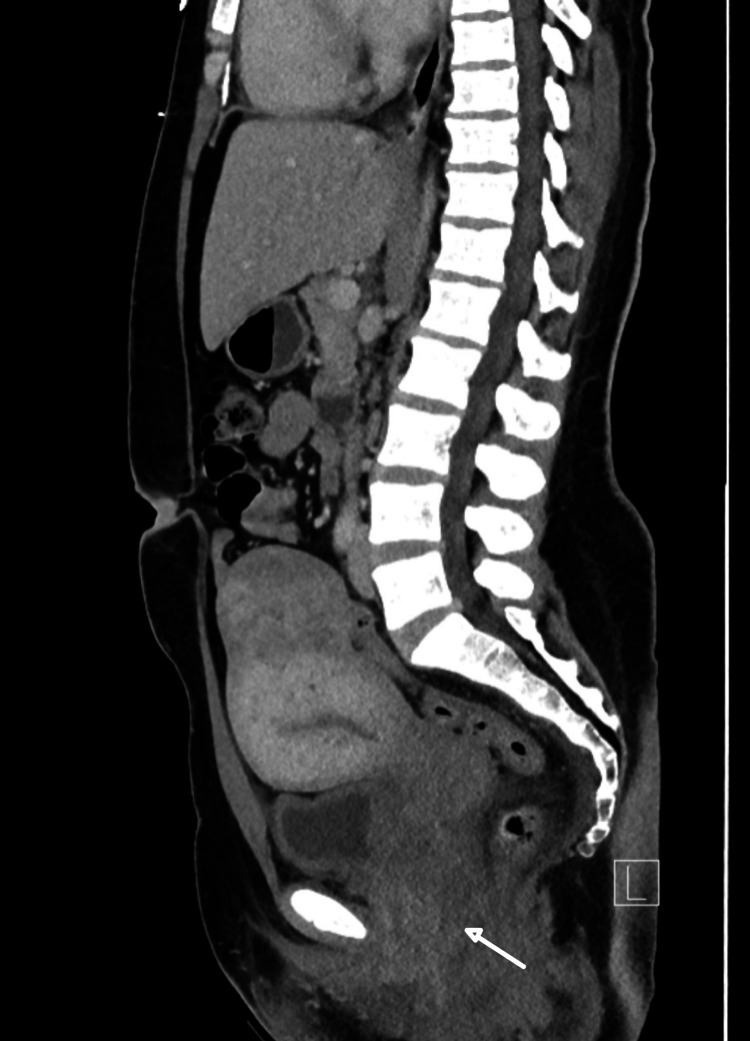
Contrast-enhanced computed tomography of the abdomen and pelvis showing vulvovaginal and cervical thickness with diffuse edema (white arrow) and findings concerning for pelvic inflammatory disease and developing an abscess

Computed tomography (CT) of the left ankle showed an avulsion fracture of the distal fibula with accompanying diffuse soft tissue edema without focal fluid collection or air. A CT of the abdomen and pelvis with contrast showed vulvovaginal and cervical thickness with diffuse edema and findings concerning for pelvic inflammatory disease and developing abscess (Figure 1).

**Figure 2:**
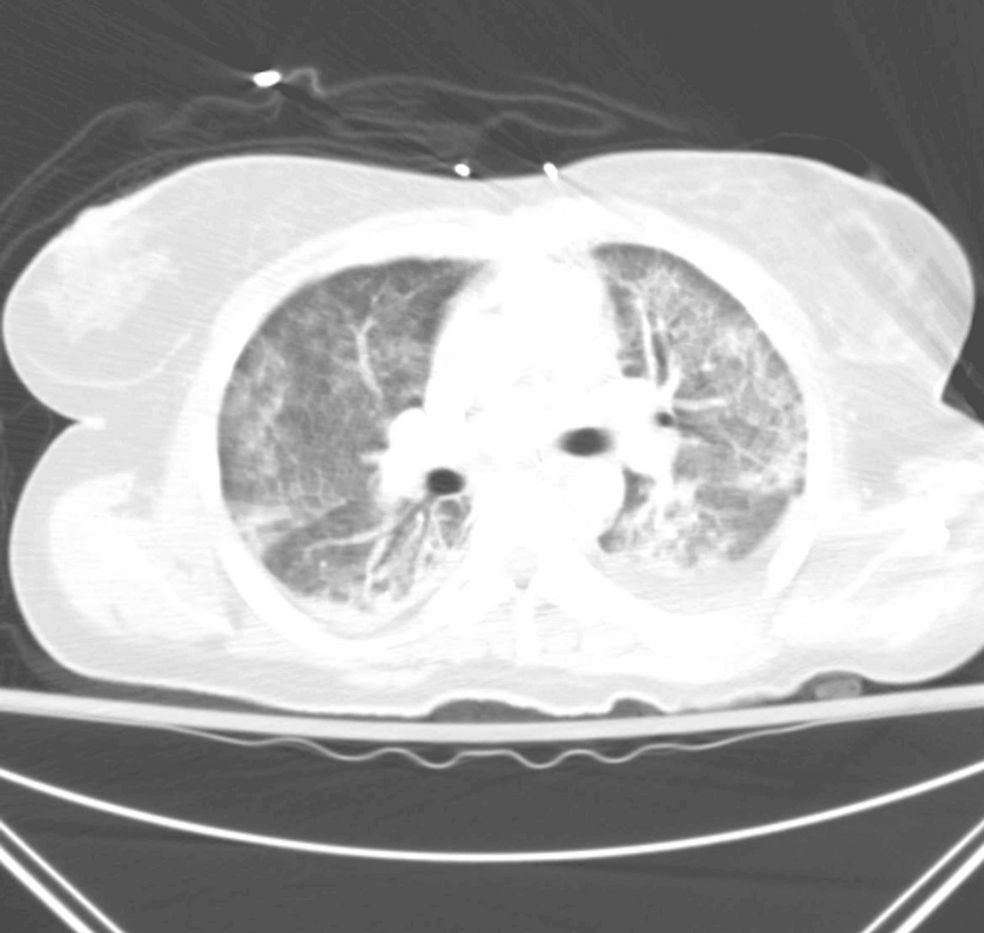
CT angiography of the chest showed bilateral multilobar disease concerning for acute respiratory distress syndrome

An initial diagnosis of sepsis with multiorgan failure was made with possible sources of infection being a septic joint, a genitourinary tract infection, or pelvic inflammatory disease. The patient was resuscitated with intravenous normal saline and started on broad-spectrum antibiotics including vancomycin and piperacillin-tazobactam. However, within two hours of presentation, she became hypotensive and tachypneic and required increased oxygen support. Arterial blood gas showed severe hypoxemia with respiratory acidosis. A CT angiography of the chest showed bilateral multilobar disease concerning for ARDS (Figure 2). A respiratory pathogen panel, including COVID-19 and rapid strep test of the oropharynx, was negative.

The patient was transferred to the medical intensive care unit (MICU) and clindamycin was added because of a strong suspicion of bacterial toxin-mediated infection. A transthoracic echocardiogram showed normal chambers and function without valvular vegetations. The joint fluid aspiration of her ankle was negative for septic arthritis. Cultures of her urine, sputum, and genitals were negative. However, her blood cultures from admission grew Group A Beta Hemolytic Streptococcus (S. pyogenes). The sexually transmitted disease panel was negative except for the presence of Trichomonas vaginalis rRNA.

Despite resuscitation, the patient’s hemodynamics and respiratory status continued to deteriorate, requiring norepinephrine infusion and mechanical ventilation. The patient met the criteria for severe ARDS. The Group A Strep cultured from her blood was resistant to Clindamycin and so her Clindamycin was changed to Linezolid. Because of ongoing fever and leukocytosis, an additional CT of the ankle with contrast was obtained and showed multiple rim-enhancing fluid collections abutting the lateral malleolus that were not present on the initial CT. These collections were aspirated and stained positive for rare gram-positive cocci and neutrophils, but ultimately cultures were negative. Similarly, a repeat CT scan of the pelvis showed multiple intervally larger fluid collections concerning for pelvic abscesses.

Because of the increasing severity of her ARDS as determined by PaO2/FiO2 (partial pressure of oxygen in arterial blood divided by the fraction of oxygen in inhaled air), the patient was placed in the prone position for two days (16 hours prone and 8 hours supine protocol), resulting in a dramatic improvement in her respiratory status. The patient was extubated after eight days. Once clinically stable, an MRI of the ankle was obtained showing continued swelling and concern for myositis while her CT of the abdomen and pelvis showed decreased inflammation and size of her abscesses. The patient was discharged home on Levofloxacin, Metronidazole, and Fluconazole for four weeks to treat pelvic inflammatory disease with pelvic abscess. On a follow-up visit at two weeks, she reported significant improvement in her pain and overall status, and a repeat CT scan of her pelvis showed improved swelling and resolved fluid collections.

## Discussion

Invasive Group A Streptococcal (GAS) infections can present in various ways, ranging from simple pharyngitis, myositis, or soft tissue infections, to bacteremia, necrotizing infections, and most severely toxic shock syndrome [5]. As currently defined by the Centers for Disease Control and Prevention (CDC), STSS requires a positive culture for GAS, the presence of hypotension, and the involvement of at least two other organs such as the kidney, liver, lung, skin, or muscle [6]. It is important to note that, based on the CDC’s current definition of STSS, organ failure is not required, just organ involvement. This allows the inclusion of desquamating rash (which often develops late) or necrotizing fasciitis/myositis as qualifying features of this syndrome.

This patient was systemically ill on presentation and eventually met the criteria for STSS (positive cultures, hypotension, lung involvement (ARDS), elevated bilirubin, and myositis). However, the source of her infection was unclear, as her presenting complaint was ankle pain that had been present for at least nine days; yet, she also had laboratory and radiographic evidence of pelvic infection. Skin or joint trauma, as well as genitourinary infections, are possible sites of entry for systemic streptococcal infection [2]. Although she presented with both, the time course suggested that the genitourinary infection was more likely to be the entry site for the present infection, as it was developing at the time of presentation and development of systemic illness while the ankle swelling had been present for at least nine days.

A CT of the left ankle revealed an avulsion fracture of the distal tibia but no evidence of necrotizing fasciitis or myositis. Joint aspiration revealed clear fluid with minimal white cells and a negative culture. A follow-up CT scan eight days later did reveal fluid collections and cellulitis surrounding the left ankle joint, but this could have been a consequence of the initial joint aspiration done on admission. Due to the patient’s improving condition, no surgical debridement was performed on the ankle. Similarly, gynecologic surgical interventions were not done due to a lack of a single drainable source. Involving a multidisciplinary team early in the disease course allowed for a prompt evaluation of both potential sources of infection. At the time of discharge and after the patient had clinically stabilized, her pelvic inflammation and abscesses demonstrated marked improvement in imaging, but she still had evidence of myositis in the ankle on MRI. Improvement of her pelvic infection coinciding with a resolution of her STSS in the absence of resolution of her ankle swelling supports the diagnosis that her pelvic infection was the source of this STSS. What is interesting about this case is the presence of these two possible sources of infection and the possibility that her ankle was seeded by circulating GAS bacteria originating from her pelvic infection.

This patient developed severe ARDS and our management was guided by two large clinical trials, the ARDSNet ARMA study, which demonstrated that low tidal volume ventilation (4-6 ml/kg predicted body weight) and low inspiratory pressures (plateau pressure < 30 cm H2O) improved survival [3], and the PROSEVA trial that showed decreased all-cause 28-day and 90-day mortality using early application of prolonged prone positioning in patients with severe ARDS [7]. Using this approach, the patient’s ARDS improved and we were able to extubate the patient eight days after intubation. In severe refractory cases of STSS, there are reports of effectively managing ARDS and cardiac instability and/or depression with veno-arterial extracorporeal oxygenation [8]. While this was considered, it was ultimately not required in this case, as the patient responded to less intensive therapy. 

In addition to source control, Penicillin and Clindamycin remain the mainstay of therapy for STSS. Clindamycin serves as an adjunct therapy in these settings primarily due to antitoxin effects [5]. It works by binding to the 50s ribosome of bacteria and inhibits protein synthesis. Due to the increased incidence of Clindamycin-resistant GAS, there is an evolving debate about whether linezolid should replace clindamycin in such cases [9]. The use of Clindamycin has been shown to decrease the presence of virulence factors in a mouse model of GAS-induced necrotizing fasciitis in both Clindamycin-sensitive and Clindamycin-resistant GAS strains, suggesting that the anti-toxin effect of Clindamycin is important even if it is not reducing bacterial counts [10]. Thus, the patient’s clinical improvement on Clindamycin despite the organism’s resistance to that antibiotic on lab sensitivities likely relates to its preserved anti-toxin function. However, despite the patient’s improvement, Clindamycin was replaced by Linezolid once sensitivities returned. Like Clindamycin, Linezolid can also block toxin production and is recommended as a substitute for Clindamycin for invasive GAS infections [11]. Of note, the sensitivity of this patient’s GAS strain to Linezolid was not tested. Current research in the field of microbiology involves investigation into the multiple virulence factors associated with Group A Streptococcal bacterium as well as the genetic mechanisms of resistance to macrolides, tetracyclines, fluoroquinolones, and sulfamethoxazole [12]. Continued investigation of antibiotic susceptibility may provide further insight into managing infections that show resistance.

## Conclusions

Group A Streptococcal infections can cause a toxic shock syndrome but have rarely been documented as originating from a gynecologic infection. Definitive treatment of GAS STSS typically involves source control through surgical intervention along with antibiotic coverage with a beta-lactam or Lincosamide. However, we were able to successfully treat our patient non-surgically using toxin-suppressing antibiotics as well as management of shock and respiratory failure. The initial diagnosis of this patient was complicated by the multiple potential sources of infection. This interesting case provided insight into managing GAS STSS with an atypical presentation. Uncertainty of the source of infection should not delay treatment, prone ventilation strategies should be considered in treating ARDS from STSS, and Linezolid showed efficacy in treating STSS with a Clindamycin-resistant GAS.
